# Exploring the Social Contributors to Biological Aging With Medical AI

**DOI:** 10.1016/j.jacadv.2024.100889

**Published:** 2024-03-20

**Authors:** Timothy J. Poterucha, Susan Cheng, David Ouyang

**Affiliations:** a Division of Cardiology, Columbia University Irving Medical Center, New York, New York, USA; b Department of Cardiology, Smidt Heart Institute, Cedars-Sinai Medical Center, Los Angeles, California, USA

**Keywords:** aging, deep learning, social determinants of health

**Figure:**
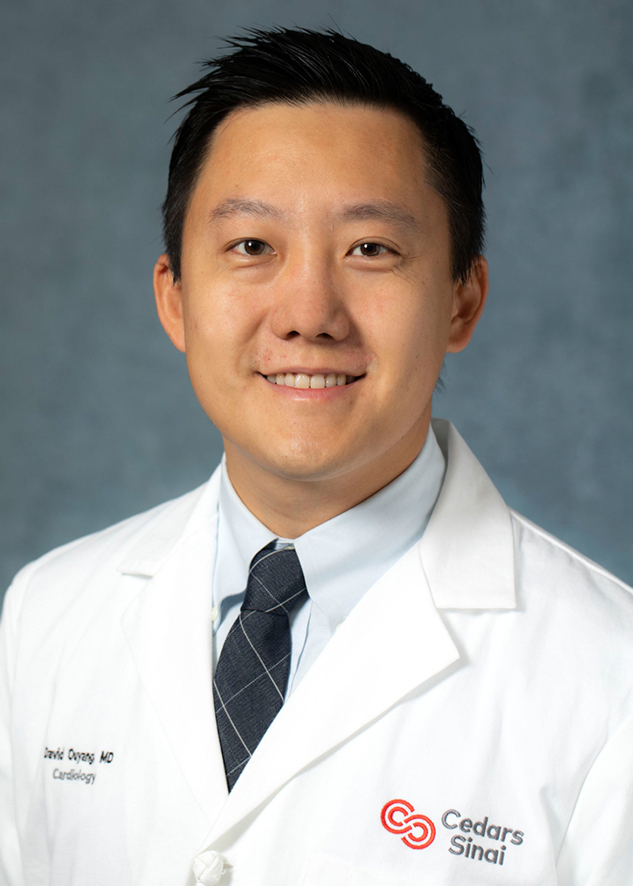

Artificial intelligence (AI) and machine learning methods have improved tremendously over the last decade, with new innovations being applied in medicine almost daily. Impressively, AI in medicine has demonstrated the ability to perform tasks that simply cannot be done by human clinicians, such as prediction of left ventricular low ejection fraction from electrocardiograms^1^ or the severity of knee pain from a plain knee x-ray. Despite the rapid advancement of medical AI, however, the black box nature of how many of these systems operate can obscure our understanding of model assumptions and, in turn, limit our ability to interpret clinical relevance and the generalizability of results.

Because AI can identify previously indiscernible patterns and relationships, an area of particular interest pertains to the social determinants of health. To this end, in this issue of *JACC: Advances*, Rajai et al^2^ have examined the relationship between social isolation and an AI-derived measure of biological aging. The premise is compelling. Could it be that social isolation, which is already known to be highly prevalent among older adults,^3^ serves to augment or accelerate biological aging? To address this question, the investigators analyzed data on social isolation collected from standardized questionnaires completed by 280,324 patients at multiple Mayo Clinic sites. These same patients also had clinical 12-lead electrocardiogram data available, and their waveforms were analyzed using an AI algorithm previously developed by the same group to estimate cardiac ‘ECG age’.^4^ The ECG-derived age estimation presumably reflects age-related cumulative stress that, when compared to actual chronologic age, can reveal a picture of either accelerated or delayed biological aging. Accordingly, the authors found that greater social isolation was cross-sectionally associated with a larger calculated ‘Age-Gap’ between estimated ‘ECG age’ and actual chronologic age. In turn, social isolation was also significantly associated with mortality risk in multivariable-adjusted Cox proportional hazards models. Strengths of the study include the vast number of patients with both validated questionnaire data on social isolation and a novel AI approach used to assess health status. The potential implications are important, given the inference that interventions aimed at reducing social isolation could slow down biological aging and, in turn, lower mortality risks.

Notwithstanding the strengths of the study, there is more work to be done. As is the case for all cross-sectional findings, social isolation and ‘Age-Gap’ associations do not imply causation. Aging, an increased burden of chronic illness, cognitive impairment, and social isolation are all tightly linked by complex, multidirectional relationships. Further work is needed to determine, for instance, the extent to which a comorbidity such as heart failure could serve as a confounder (eg, social isolation leads to undertreated risk factors and then subclinical heart failure that alters the ECG) or a collider (eg, while social isolation leads to undertreated heart failure risk, ECG traits reflect cardiac remodeling that also predisposes to heart failure). These classic conundrums of confounding and collider effects are admittedly prominent issues in training medical AI models.

Fortunately, lessons from the past can offer guidance on steps for the future. When limited to cross-sectional data, as is often the case for the development and application of many AI models, conventional mediation analyses may lend some insights regarding what proportion of the variation in an outcome (eg, the ‘Age-Gap’) is attributable primarily to a given predictor (eg, social isolation) vs mediated by other variables (eg, comorbidities). Similarly, in prospective analyses, the extent to which the observed relationship between social isolation and mortality risk is mediated by confounders could be formally evaluated.^5^ Table 4 in Rajai et al’s^2^ manuscript confirms that the ‘ECG age’ construct aligns more closely with cardiovascular than noncardiovascular comorbidities. Incorporation of additional noncardiac data may enable optimization of algorithms that more broadly estimate biological age in a manner that could be even more strongly linked with social isolation. Ideally, future work aimed at clarifying the directionality of the observed associations will involve examining their temporality, including changes over time in the predictor and the outcome. In this regard, we are reminded of the seminal social network study by Christakis and Fowler that reported from over 30 years’ of longitudinal data how progressive weight gain and development of obesity in 1 person was significantly associated with the same among friends, siblings, and spouses.^6^ Accordingly, studies validating the initial findings from Rajai et al could in the future demonstrate that chronic social isolation is not only associated with excess biological aging but with an increasingly widening gap between biological and chronologic age even after accounting for concomitant and progressive comorbidities.

The extraordinary promise of what AI can offer clinical medicine will require extraordinary efforts to ensure rigor, reproducibility, and relevance. Rajai et al are to be commended for recognizing the importance of tackling important and yet elusive questions regarding the social drivers of health and disease. With the advent of increasingly powerful AI methods, the potential to solve some of the persistently difficult problems in medicine is within grasp. That said, the greater the scope and complexity of the problem being addressed, the greater the need for care and attention to every aspect of the approach—from quality of data capture to balance in data structure, handling of covariates, model assumptions, and validation techniques. Despite the appealing concept that AI could be a 'magic pill' for many ailments, physician scientists have long understood that gaining new knowledge that will stand the test of time in practice will still require grounding in the experimental method and, ultimately, the benchmark truth provided by rigorous prospective clinical studies.

## Funding support and author disclosures

Dr Poterucha owns stock in Abbott Laboratories and Baxter International with research support provided to his institution from the Amyloidosis Foundation, American Heart Association, Eidos Therapeutics, Pfizer, and Edwards Lifesciences. Dr Cheng has received consulting fees from UCB and Viz.ai; and research grants from 10.13039/100000002NIH R01-HL131532 and 10.13039/100000002NIH R01-HL142983. Dr Ouyang reports consulting fees from AstraZeneca, Ultromics, EchoIQ, and InVision; and research grants from 10.13039/100000002NIH K00-HL157421 and Alexion Pharmaceuticals.
